# American Dental Convention

**Published:** 1862-09

**Authors:** 


### Proceedings of Societies.

### AMERICAN DENTAL CONVENTION.

### EIGHTH ANNUAL MEETING.

### REPORT OF PROCEEDINGS AND DISCUSSIONS.

BY T.

THE American Dental Convention met at Trenton Falls, August 5th, 1862. The President, Dr. Jno. Allen, in the chair. The other officers, except the Treasurer, were present. Dr. T. B. Whitney was chosen Treasurer *pro tem.*

The following members of the profession were present, enrolled their names and paid dues, (The assessment was one dollar per member) viz:

| NAME. | RESIDENCE. | |
|---|---|---|
| Dr. T. B. Whitney | Buffalo, | N. Y. |
| Dr. F. H. Clark | New York, | N. Y. |
| Dr. W. B. Roberts | " | " |
| Dr. John Allen | " | " |
| Dr. W. H. Dwinelle | " | " |
| Dr. F. H. Norton | " | " |
| Dr. W. H. Atkinson | " | " |
| Dr. T. H. Burras | " | " |
| Dr. W. B. Franklin | " | " |
| Dr. G. Chevalier | " | " |
| Dr. Chas. S. Miles | " | " |
| Dr. A. Jones | " | " |
| Dr. N. W. Kingsley | " | " |
| Dr. W. H. Allen | " | " |
| Dr. E. F. Willson | Rochester, | " |
| Dr. J. A. Perkins | Albany, | " |
| Dr. L. W. Rogers | Utica, | " |
| Dr. G. B. Snow | Hornelsville, | " |
| Dr. A. Westcott | Syracuse, | " |
| Dr. M. French | Cortland, | " |
| Dr. M. Tefft | Cambridge, | " |
| Dr. Thos. D. Evans | Utica, | " |
| Dr. W. H. Klock | Little Falls, | " |

| NAME. | RESIDENCE. | |
|---|---|---|
| Dr. S. M. Robinson | Watertown, | N. Y. |
| Dr. J. C. House | ———, | " |
| Dr. H. A. Coe | Theresa, | " |
| Dr. Chas. F. Mennier | Brooklyn, | " |
| Dr. N. D. Ross | Troy, | " |
| Dr. Geo. E. Hayes | Buffalo, | " |
| Dr. E. P. Hadcock | Newport, | " |
| Dr. Chas. W. Shapley | Utica, | " |
| Dr. J. C. Cowles | Rome, | " |
| Dr. A. N. Priest | Utica, | " |
| Dr. J. S. Brown | Green, | " |
| Dr. A. W. Kingsley | Elizabeth, | " |
| Dr. W. O. Laird | Stittsville, | " |
| Dr James Brown | Rochester, | " |
| Dr. L. E. Brockway | Utica, | " |
| Dr. H. Jameson | Lyons, | " |
| Dr. S. B. Palmer | Tully, | " |
| Dr. W. B. Hurd | Brooklyn, | " |
| Dr. E. L. Swartwout | Utica, | " |
| Dr. D. L. Golday | Oswego, | " |
| Dr. W. C. Parks | Brooklyn, | " |
| Dr. G. N. Foster | Utica, | " |
| Dr. L. W. Bristol | Lockport, | " |
| Dr. Jno. L. Clark | Waterloo, | " |
| Dr. W. C. Tremain | Rome, | " |
| Dr. S. F. Tremain | " | " |
| Dr. M. B. Stetson | Prospect, | " |
| Dr. S. Mapes | Fishkill Landing, N. Y. | |
| Dr. E. M. Skinner | Syracuse, N. Y. | |
| Dr. Wm. A. Pease | Dayton, O. | |
| Dr. Corydon Palmer | Warren, O. | |
| Dr. J. Taft | Cincinnati, O. | |
| Dr. F. Searle | Springfield, Mass. | |
| Dr. J. C. Robbins | Jersey City, N. J. | |
| Dr. G. F. J. Colburn | Newark, | " |
| Dr. G. W. Ellis | Philadelphia, Pa. | |
| Dr. J. R. M'Curdy | " | " |
| Dr. T. L. Buckingham | " | " |
| Dr. F. N. Johnson | " | " |
| Dr. Chas. H. Neal | " | " |
| Dr. S. Driggs | Lexington, Ky. | |
| Dr. D. W. Stone | " | " |
| Dr. J. H. Smith | New Haven, Conn. | |
| Dr. J. T. Metcalf | " | " |
| Dr. A. Hill | Norwalk, | " |
| Dr. C. G. Sumner | " | " |

| NAME. | RESIDENCE. |
|---|---|
| Dr. B. Wood | Indianapolis, Ind. |
| Dr. C. F. Campbell | Murfreesboro, N. C. |
| Dr. C. A. Hastings | Rio de Janeiro, Brazil, S.A. |
| Dr. Jas. Lewis | Burlington, Vt. |
| Dr. J. W. Ellis | Chicago, Ills. |
| Dr. E. A. Bogue | " " |
| Dr. D. W. Perkins | Milwaukee, Wis. |
| Dr. J. Bower | Ingersoll, Canada West. |
| Dr. J. B. Willmott | Milton, " |
| Dr. E. Beckwith | ———, ———. |

After the enrollment, the constitution was read by the Secretary.

On motion of Dr. Rogers,

*Resolved*, That Dr. F. H. Norton be employed to report the proceedings of this Convention for publication in the Utica papers, and that he be paid $4.00 per day for the same.

The Minutes of the last Cenvention were read, corrected, and approved.

The Corresponding Secretary gave a summary of the business transacted by him since the last meeting. Among other things, he read a letter of invitation, issued by him, to the dentists of Canada, to attend this Convention, and participate in its proceedings. This invitation received the hearty concurrence of all the members of the Convention.

A resolution, embodying a standing invitation to all the dentists of the American continent to attend the future meetings of this Convention, was adopted.

The committee on the subject of Army and Navy Dentists reported that they had not accomplished anything definite.

A communication was received and read, from Surgeon General Hammond, U. S. A., addressed to Dr. J. D. White, a member of the committee. In that letter he expressed his most unqualified approval of the introduction of dentists, as such, into the army and navy; he considers it an important matter, and made some suggestions in regard to the method of accomplishing it.

Dr. Taft, in referring to the matter, said that he had been several times applied to by officers, either to go, or send good operators to the army; had been promised protection and assistance by the officers; had also been importuned to the same effect by surgeons. These requests came with the statements that the services of dentists are much needed. From such connections great advantages would accrue, in two ways: 1st. Benefit to the soldiers, and 2d. Benefit to the profession, in the recognition it would thus obtain.

On motion, a resolution of thanks was tendered by the Association to the Surgeon General for the interest manifested.

On motion of Dr. Westcott,

*Resolved*, That each member of this Convention be authorized and requested to labor for the promotion of this object. (And we would further suggest that every one give a statement of his efforts and the result to the committee, at the earliest practicable period.—Rep.)

The committee on army and navy dentists was continued, with the addition of Dr. T. H. Burras. It consists of W. H. Atkinson, J. D. White, Geo. H. Perrine, W. B. Franklin, I. J. Wetherbee and T. H. Burras.

The election of officers was the next order of business.

Drs. Rogers and Whitney were appointed by the Chair to act as tellers.

Upon vote being taken by ballot, Dr. A. Westcott was found duly elected *President* for the ensuing year.

J. Taft, *Vice-President.*

W. B. Roberts, *Corresponding Secretary.*

F. Searle, *Recording Secretary.*

J. C. Robbins, *Treasurer.*

During the balloting, on motion of Dr. Westcott,

*Resolved*, That in balloting for officers, one of the three highest nominees withdrawing, the next highest shall be substituted.

On motion,

*Resolved*, That the subsequent sessions of this Convention meet at 9½, A. M., and continue to 1, P. M. Afternoon session meet at 3½ o'clock, and continue to 5½.

The Convention now adjourned to 9½ o'clock to-morrow morning.

(There was no afternoon session held, the house being occupied by the funeral of the man of whom it was obtained.—Rep.)

SECOND DAY.—WEDNESDAY, AUG. 6TH.

Convention met according to adjournment, Dr. Allen in the chair.

By invitation, a large number of ladies were present at this session.

The Secretary being absent, Dr. T. H. Burras was appointed Secretary *pro tem.*

The Minutes of the former session were read, amended and approved.

The roll was called by the Secretary, almost all the members answering to their names.

The Treasurer presented his report, which presented the following exhibit, viz.: The receipts for the last year were, from former Treasurer and from payment of dues, $191 47. Disbursements during the same time, $150 70, leaving a balance on hand of $40 77.

The President appointed Drs. F. H. Clarke and D. W. Perkins a committee to audit the Treasurer's account.

The retiring President, Dr. J. Allen, delivered a very interesting address, which was replete with instruction and advice, and was received by the audience with almost breathless attention.

(It will be found on page 394, present number of the Register.—Rep.)

Drs. Rogers and Stone were appointed to conduct the President elect to the chair, when he made the following remarks:

He had hoped to come here merely to brush up, and receive what might be presented, and to aid in a quiet way the objects contemplated by this Convention. He did not come to make speeches, nor to take a prominent position. This meeting calls up in memory a host of recollections ; gladly would he present them, but time and circumstances forbid.

This body is composed of volunteers; we have no drafted members. This is pleasant; nevertheless there is great cause for regret—our Southern brethren are not with us. These things depress us, and the accompanying circumstances have kept us from pursuing the science of our profession. But such a number as are here to-day gives us new courage, and inspires us with hope for the future.

The first meeting of the old American Dental Association, held twenty years ago, comes fresh to my memory. The principles upon which it was based are correct, and are as true and applicable to-day as then. The declaration of that Association was, The *advancement* of *dentistry* and the *suppression* of *quackery*. The manner of his introduction into that society made an impression upon his mind that had never been eradicated ; he remembered the close, *searching* examination, occupying two hours, at the hands of such men as Hayden, Harris and Parmley, and after his admission, the warm, brotherly grasp of these men inspired him with an energy and resolution that have been as a firm rock to him ever since. It gave a great impetus to his progress; he resolved to *do* and *be*.

By associated effort we are made to *go*.

The anticipation of these meetings always afforded him much pleasure. He permitted no ordinary obstacle to prevent his attendance. He would be pleased to read to this Convention the Preamble and Constitution of that Association. The prominent ideas held forth were, "The elevation of the profession, and the suppression of quackery."

Many consider dentistry as a trade; they *call* it a trade, and *think* it a trade. If it is no more than this, we had better adjourn immediately and go home. There are many sciences of which the true dentist must be the possessor; and a knowledge of science generally will not injure a dentist. The distinction between a trade and a profession should be clearly defined and fully understood. The professional man *must* have literary and scientific attainments. The artisan may not possess either, and yet the dentist must be an artisan, as well as a scientifician and a man of letters. The speaker here referred to the wide range of mechanical manipulations which the dentist is called upon to perform. We should be stimulated to go on in every department. Individual effort must be constantly kept up, and associated effort always when opportunity affords. Associated effort builds our railroads; builds telegraphs, and sends missionaries to the heathen. He hoped the elevation of the profession and the suppression of quackery will be the object and work of each and every member of this Convention.

Break down cliques; destroy party spirit—set up a high and brilliant mark for your aim—do not permit personal interests to come in. He tendered thanks to the Convention for the honor conferred, and asked assistance in the discharge of duty.

Dr. Dwinelle—Concurs fully in the remarks of the President. Twenty years ago, the profession received a large bequest from our predecessors. We owe much to them; should endeavor to pay the debt; we should endeavor to execute the good and just declarations which they made. Our profession has grown so as to challenge the recognition of the world. In order to retain this, we must retain our present status, and not stand still either. He then offered the following preamble and resolutions, and moved their acceptance and adoption by this Convention:

PREAMBLE.

Whereas, The dignity and good name of our profession has been assailed, in times past, by those who would prostitute the honor of our noble calling to base uses; and

Whereas, Through an almost criminal weakness and supineness on our part, we have, unwillingly, been made the instruments of increasing this evil until it has reached an alarming extent; and

Whereas, This evil has shown itself, especially of late, in the countenance and aid that has been given to the practice of forcing upon us the use of certain patent rights, sometimes by threats, until tribute is paid, and sometimes, upon influential members, by presentation. Patents upon articles either worthless in themselves, or by right of prior invention, belonging to the profession, or patents in themselves otherwise invalid; and

Whereas, Our reproach is being daily augmented by these practices until we are obliged to rise in our might to protest against it, and prevent any further disgrace. Now, therefore, be it

*Resolved*, by the American Dental Convention, representing the whole body of the profession throughout the country, That it is our duty, in view of these facts, to here, and at this time, in behalf of those we represent, strongly and firmly protest against all such action as we have mentioned, or shall hereafter discover, as dangerous, fraudulent, and dishonorable, and that the system of patents, when thus prostituted to nefarious purposes, is a reproach, from which the profession must be purged.

*Resolved*, That we do endorse all action that has been or may be taken, by any individual or body, in our profession, having for its basis such protection of the standing and interests of the same, and such reprobation of all that is injurious and unjust to them in this matter.

The preamble and resolutions were accepted and adopted, as written—a motion to lay them on the table being voted down; the previous question being moved by Dr. Rogers, they were passed, without debate, by a vote of 29 to 20.

On motion, a resolution of thanks to the retiring officers of the Convention was adopted by the meeting.

On motion, the incoming officers were introduced to the Convention, and took their seats.

On motion,

*Resolved*, That we now proceed to the regular order of business.

Anæsthetics being the subject,

Dr. Rogers— Opened the discussion. For producing anæsthesia prefers chloroform; has used it in two thousand cases; never had any unfavorable symptoms except in two cases; in one, numbness in one arm, which passed off after a time; in the other there was dizziness, headache, and unfavorable symptoms for a considerable time. His experience is decidedly in favor of chloroform; thinks he can administer it better than it is usually done by the general surgeon.

In regard to the administration, 1st. Quiet the patients as much as possible; imbue them, if possible, with a feeling of security; can always give a second time with much better results. He always places the patient in the proper position for the extraction of the teeth. Oil the lips, that they may not be cauterized by the chloroform; put a wet cloth on the head, to counteract cerebral excitement; then hold the nose tightly—this will cause the patient to open the mouth, thro' which the agent should be inhaled; he is governed in regard to the rapidity and amount inhaled, by the breathing of the patient; occasionally asks him quietly if he has enough, when he answers slowly and carelessly, then lance the gum, with the napkin still in the mouth; if there is no pain, then extract. He uses nothing for restoration except ammonia, or something of the kind.

Dr. Colburn—Considers this a very interesting subject; has condemned chloroform and used ether. Obtained Morton's patent for the use of ether, and has used it upon an average once a day since.

After the introduction of chloroform, used it about nine months, till the death of a lady from its use in Cincinnati; then abandoned it. He referred to many cases of death from chloroform, of which there have been a large number. Fright of the patient tends to produce alarming symptoms; he usually administers ether upon a napkin, placed in a concave sponge; the indication of the proper condition is when the eyelids cease to quiver, or are at perfect rest; then operate.

Dr. Kingsley—Is quite familiar with this subject; is well acquainted with the surgeons in New York. One class of physicians gives only ether, another gives chloroform with entire freedom. Ether exhilerates, so much so as sometimes to be annoying. To obviate this, when the patient takes the chair, gives him strict charge not to speak; let this be the last impression on the mind; in no case where this is done, is there any talking or boisterous manifestations; the patient is perfectly controllable.

Dr. Dwinelle—Some prefer ether, others chloroform. In this country ether is more used than chloroform; he thinks the difficulty arising from the use of chloroform depends upon the method of administration, rather than upon the chloroform. He prefers ether for a long operation, but usually uses chloroform. Always leave upon the mind of the patient a proper impression—the last thought is the ruling thought; the operator can and should give this. Chloroform should be given on an empty stomach.

Prefer to have the patient in almost an erect posture, to prevent closure of the epiglotis, and to inhale through the mouth till relaxation of the muscles takes place. Touch the eyelids till they will not respond to the contact. When there is a temporary cessation of breathing, *tells* the patient to breathe, insists upon it, and if they do not respond, then resorts to artificial respiration, irritates the epiglotis. He gives chloroform in all kinds of cases. Thinks both ether and chloroform good, but uses either as seldom as possible.

Dr. Hayes—Applies chloroform locally; would not use chloroform for a considerable time,—a year or more,—but was afterwards forced to it by the urgent demands for it; have been using it locally, not to entire insensibility, but it mitigates the pain very much.

Dr. Ellis—Advises all his patients to have their operations performed without anæsthesia; uses ether only, and that very seldom. There is no safe way for a man to part with his senses and sensibilities. Surgeons admit that they have killed patients with chloroform.

Dr. Whitney—Concurs with Dr. Ellis. For the administration of ether, folds a napkin funnel shape, that will embrace both the nose and mouth; into this pours the ether, and applies that it may be inhaled both by the mouth and nostrils.

Dr. Foster—Uses chloroform and ether equal parts, but always with great caution; will not use chloroform; does not give to the extent that many do; always throws the responsibility upon the patient as far as possible. Applies it to the gums for local anæsthesia.

Dr. A. S. Perkins—Asked if death was not liable to occur in many cases.

Dr. Dwinelle—Doubts if death ever occurred from chloroform when proper care was taken.

Dr. Allen—The cause of the ill effects of anæsthetics is not well understood. He referred to and gave a description of the Cincinnati case—was present soon after the accident; he thinks chloroform more dangerous, because it is more volatile and subtle than ether; the blood is thrown upon the brain, and by pressure its function is arrested, and death occurs.

Dr. W. B. Roberts—Uses chloroform; begins its administration very cautiously, and watches its effects closely; lets the patient sit erect; simply folds up the napkin, and uses the chloroform upon it; he thinks Dr. Whitney's method of preparing the napkin not good, as it would not admit a sufficient admixture of air.

Adjourned till 3½ o'clock, P. M.

SECOND DAY.—AFTERNOON SESSION.

The Convention was called to order by the President at 3½ o'clock. The discussion on chloroform was resumed.

Dr. Rogers—Does not give chloroform for one or two teeth. In taking out a considerable number of teeth, the shock is very great to the nervous system, but under the influence of chloroform, it is scarcely perceived, and the chloroform gives no shock. In regard to the deaths recorded, they seem to be greater in number than they are, for many of them are recorded over and over again. He thinks that the majority of deaths have resulted from carelessness or abuse of the agent, so that in reality these fatal cases do not constitute an argument against its use.

Dr. J. A. PERKINS—Used chloroform in the beginning, but owing to some fatal results occurring from its use, abandoned it; he thinks death may occur in any case, even though it may have been administered before. He thinks that those who advocate its use so strongly stultify themselves; they say it is perfectly safe, and yet discourage its use, and advise great caution. He abandoned its use six years ago.

Dr. BOGUE—Does not use chloroform, because it is too much trouble. Thinks that statistics show that death is less frequent in large operations, where chloroform is used, than where it is not. Death from the use of chloroform is occasioned by simple asphyxia.

Dr. D. W. PERKINS—One effect of chloroform on the blood is an enlargement of the red corpuscles. Chloroform produces insensibility by operating especially on the medulla oblongata. The nerves of sensation are more superficial than those of respiration; the latter are deeply seated; if these are fully narcotized, then life becomes extinct. He thinks chloroform the greatest benefactor of the age. At the time of the administration, he always endeavors to imbue the patient with the utmost confidence. If the breathing is good, all is well. For a test, requires the patient to hold up the hands as long as he can, the elbow resting upon the arm of the chair, and when it drops, operates. Other tests may also be employed, as cutting the gum or pinching it with a nail. He thinks ether is quite as dangerous as chloroform, if carried to the same extent. With chloroform a few minutes is time enough to produce the desired condition; in operating, keep the blood from going down the throat.

Dr. B. W. FRANKLIN—If there is a possibility of the occurrence of death in one case in ten thousand from the use ef chloroform, he would not use it; but ether can be used without danger; he has taken ether and chloroform a great many times; would not have even a small operation performed without it; he uses it largely; ether may be washed till it is perfectly pure, and as pleasant as chloroform, and about as efficient; its offensive odor is thereby removed. The method of washing is very simple—take a glass bottle, bore a small hole just at the bottom, make a little wooden pin to stop it; then put in the ether with as much or more pure water, shake well, and let it stand a moment; take out the plug, and draw off the water, it being at the bottom; this may be repeated until the ether is perfectly pure.

Dr. DWINELLE—It is important for us to consider how many lives have been saved by the use of anæsthetics.

Dr. C. PALMER—Uses chloroform and ether mixed; uses it only where there is one or two teeth to be extracted; but for a long operation, prefers to have the patient without anæsthesia, as we can then have the intelligent coöperation of the patient; while under the influence of anæsthesia, the patient resists.

Dr. M. B. STETSON—Have used chloroform successfully for five years; does not push it to entire insensibility; death is usually produced by asphyxia, the epiglottis being closed.

Dr. TEFFT advocates the use of chloroform as a local anæsthetic.

Dr. ATKINSON—Thinks Dr. Perkins is rather hypothetical. How does he know that the red globules expand, and that certain changes are made in just four seconds? Thinks death is caused by closure of the epiglottis. If a patient becomes uproarious with ether, just give a few inhalations of chloroform, and that will quiet all down nicely.

Dr. A. W. KINGSLEY gave a case of a woman who inhaled ether, and did not speak for six hours afterwards; applied ice to the head for thirty-six hours. She has suffered more or less in the head ever since.

Dr. F. SEARLE referred to a military surgeon who was at one time very cautious and fearful in regard to the use of chloroform; he became acquainted with Dr. Simpson, and was relieved of all his fear, and has used it since extensively —in many thousands of cases, without any bad results. He (Dr. S.) gives chloroform very slowly; does not call a physician, to avoid responsibility; knows more about chloroform than physicians usually do; will not let a physician control him. He administers chloroform by applying a vial containing it to one nostril, having the other and the mouth closed, being careful to admit a sufficient amount of air.

Dr. Westcott—Thinks it is best to do without chloroform when it is at all admissible to do so. Has had one alarming case, which was quite a caution to him. Any careful, judicious operator will feel his way, and go slowly and safely on, and in this way accomplish the object without much liability to danger. His test is for the patient to hold the fore-arm erect, the elbow resting upon the arm of the chair; when the hand drops, then operate.

Dr. Burras—Upon its first introduction, used chloroform very freely, and with great avidity; administers by the vial, as directed by Dr. Searle; uses the uplifted hand test. He thinks the semi-conscious state is as far as it is necessary to go, for dental operations; advises not to use chloroform when it can be avoided; uses it frequently for producing local anæsthesia.

Dr. John Allen—Uses chloroform, tincture of arnica and aconite, for producing local anæsthesia; applies on a pledget of cotton directly to the gum, and in many cases with good success.

Dr. Hurd—Thinks chloroform is very good in its place, but is not admissible for extracting teeth; he does not use it; thinks if the patient is calm, and can control himself, he is far better without any anæsthetic.

Dr. D. S. Goldey—Had used ether and chloroform, but had abandoned it since the death of a neighbor, who called to have him administer chloroform; he refused; the man went to Toronto for chloroform, took it, and died. You perhaps all remember it as the McChesney case.

Dr. Coe—Had used chloroform several years without injury, but have recently used it to produce local anæsthesia; referred to the galvanic current, as applied in extraction,—operates well in some cases. Chloroform should not be used as a local application for the extraction of the wisdom teeth, for in some cases, running back upon the fauces and into the throat, the mucous membrane of these parts would be cauterized thereby.

Dr. Buckingham—Thinks there are no well authenticated cases of death from ether; thinks the action of chloroform similar to that of ether.

Dr. Hill—Thinks that the continued use of ether and chloroform an evil, both to patient and operator, and will not use it; thinks it unnecessary, and many a time indirectly injurious; referred to two cases of death that occurred at New Haven.

The President announced the following as the Executive Committee for the ensuing year, viz.: Drs. W. H. Dwinelle, T. L. Buckingham, W. D. Stone, Wm. A. Pease, D. W. Perkins.

Adjourned to 9½ o'clock to-morrow morning.

THIRD DAY—9½ O'CLOCK, A. M.

Convention met according to adjournment, President in the chair.

Minutes of yesterday's proceedings were read by Dr. Burras, Secretary *pro tem.*, corrected and adopted.

The committee appointed to audit the Treasurer's account reported that they had examined the same and found it to be correct.

According to the regular order of business, half an hour was appropriated to miscellaneous business.

Mr. G. Chevalier exhibited an instrument for heading pins, in teeth or blocks, designed for rubber work. It is a good instrument, and accomplishes its work well. (The pin-head rivets, we think, are always preferable to the common pins bent.—REP.)

Dr. B. W. FRANKLIN exhibited a vulcanizer, with a new and beautiful attachment for the head; this attachment consists of five collar screws, passing through taps, arising from the upper surface of the top near its circumference; the screws run diagonally with the plane of the top. By this arrangement the screw collars are brought to bear upon a flange or rim at the top of the heater. By tightening the screws, the top closes firmly on to the heater.

Dr. B. W. ROBERTS exhibited for E. A. L. Roberts a fusible gauge, embracing, so far as we could see, about the same principle as Franklin's fusible gauge; also sections of seven teeth each, or half an arch. These in many cases would be very desirable, especially where there is a smooth, regular arch, are perhaps a little more difficult to articulate.

Dr. GEO. E. HAYES exhibited a vulcanizer with a new head attachment; there is a double head with set screws. It seems to be a very efficient arrangement.

Dr. WHITNEY exhibited a vulcanizer, the heater of which is in two pieces; he uses black lead on the packing to prevent sticking.

Dr. B. WOOD, by request, made some interesting remarks on alloys, in reference to change of proportion by fusion; he suggested that heat merely to the point of fusion is not sufficient, ordinarily, to change the proportions of alloys, to any appreciable extent at least.

On motion,

*Resolved*, That a committee of five be appointed to nominate places for the next annual meeting.

The committee consisted of Drs. Whitney, Taft, M'Curdy, Stone and Foster.

The next subject for discussion, viz.: alveolar abscess, was now taken up.

Dr. ATKINSON—Abscess involves inflammation; this includes molecular degeneration, and this an elaboration of plasm; if this could be removed, union by first intention would be the result. If the alveolar process is removed, it is none the less an abscess.

*Treatment.*—Remove the dead bone, leaving the periosteum, which will elaborate a plasm, which will form bone, and the surrounding tissues will be restored to health. He gave a case illustrative of his treatment. In treatment he employs the following agents: creosote, iodide of mercury, hydridate of potash, and iodine. For iodine dressings, use a gold or platinum probe. For syringing out abscesses, uses tincture of arnica one part, and water four parts, then introduces diluted creosote.

For treatment of an abscess for central incisor, open the passage freely with a bistoury; remove all necrosed bone and every obstruction, and insert pledget of cotton, charged with dilute creosote. In cases where abscesses are developing, diagnose correctly, then if the knife is used, cut right down to the point of the fang, drawing the knife down a little after it is introduced; this relieves the pain; then wash out well; then dry dress once, then with creosote; tannin and glycerine makes a good dressing—two parts of the former to one of the latter.

The Convention now took a recess of five minutes. Business being resumed, on motion,

*Resolved*, That the selection of a place for the next annual meeting be the order of business for 3½ o'clock this afternoon.

Dr. TAFT referred to the reproduction of bone being practicable; referred to the removal of dead bone by two methods, viz: by an operation and by absorption; thinks the periosteum usually dies with the bone, or soon after, and if so, can the bone be reproduced? If periosteum will elaborate bone material and form bone, will not a plasm be produced, out of which periosteum will be formed, and will the tissue beyond be reproduced upon the same principle, when there is a starting point remaining? Referred to the special action of various remedial agents. Creosote forms a compound that serves as a covering or protection to the affected part. It perhaps also stimulates the parts to a more healthy and vigorous action, causes a freer circulation, and in this way relief to the congested vessels is produced. Also referred to the action of iodine—is a good counter irritant; increases the action of the absorbents.

Gave the history of a case of alveolar abscess and its treatment. It involved the six anterior teeth, and was a very bad case; it was successfully treated upon the principles already suggested. (The description would be too prolix for this report.—Rep.)

Dr. Westcott—Has done much in the way of treating alveolar abscess; has treated with about the same results and in much the same way as Drs. Atkinson and Taft. Removes every obstruction; fills the fang in the early part of the treatment, and then makes the applications for the cure of the abscess through the opening in the gum.

In the beginning of the treatment, preferred to open the canal through the fang perfectly, and inject through it into the abscess diluted creosote. Does not think that the surrounding tissues can be made to adhere to the fang of a tooth after it has been divested of its periosteum, especially after the root has been cut or scraped.

But after all is done that can be done, we have only succeeded in partially restoring the normal condition, and there is great liability of the return of active disease.

Dr. Atkinson—Periosteum will remain attached to dead roots.

Dr. Stone—We do not hesitate to endeavor to restore diseased parts, even though we can not perfectly bring back the original condition; if we can't do everything, let us do as much as we can. Does not use a syringe, but uses a probe with cotton upon it, upon the principle of a force pump, and with this drives creosote through the fang and abscess Always uses creosote in the fangs of dead teeth before filling.

Referred to a case of abscess after a tooth was filled; treated it through the gum with creosote successfully, without removing the filling. Gave a case of alveolar abscess, terminating by a fistulous opening through the cheek; quite a depression was made upon the cheek by adhering to the gum. In the treatment this attachment was cut, and a pledget of oiled cotton placed in so as to hold the part out to its proper position. It was retained so till the parts healed; then all was well, and the cheek had its natural shape. He wishes to know what is the cause of these depressions.

Adjourned to meet at 3½ o'clock, P. M.

AFTERNOON SESSION—3½ O'CLOCK, P. M.

The Convention was called to order by the President.

According to resolution, the selection of a place for the next meeting of the Convention was the order of business. The committee on nominations reported the following places, viz.: Saratoga, Atlanta City, and Bedford Springs. After considerable discussion, Saratoga was selected as the place of the next annual meeting of this Convention; and the first Tuesday of August, 1863, at 10 o'clock, A. M., as the time of meeting.

On motion,

*Resolved*, That the third particular of the first division of the programme of subjects be passed without discussion. The second division was also passed.

*Resolved*, That all papers or essays that shall be sent to this Convention by members or others shall be referred to the Executive Committee, and disposed of by their direction.

The diseases and treatment of dental pulp was then taken up for discussion.

Dr. SEARLE—Covers exposed pulps with oxy-chloride of zinc—a thin layer, then fill with gold; the tooth may ache for a short time, but it soon subsides. It is easily applied by coating it on a thin cloth, and laying this on the exposed point; has filled a great many within the last year, and has lost but one, that he is aware of.

Dr. Colburn—Thinks he has used os-artificial more than any one else; thinks it a good preparation for filling; also to be used as suggested by Dr. Searle. It does not, under ordinary circumstances, destroy the vitality of the teeth.

Dr. Westcott—Had at one time capped nerves rather extensively; thinks there are many things about alike good for capping nerves. Nerves die under all caps usually; they may not immediately, but sooner or later that will be the result. In his hands, that treatment of nerves has not succeeded.

Dr. Rogers—Asks for facts; seldom attempts to cap or even to save a nerve after it is exposed. We do not want exceptional cases, presented at least as data upon which to base a mode of practice. Is this treatment of pulps sufficiently successful to justify us in adopting it as a practice? Have not used oxy-chloride of zinc. Thinks, as a general thing, a nerve can not be disturbed and live; arrives at this conclusion from the nature of its structure, and from the peculiarity of its location; together with experience.

Dr. Roberts—Always when there is hope of saving a pulp, endeavors to do so; takes into consideration the condition of the pulp, the time which it has been exposed, and the size of the orifice of exposure. He is convinced that the orifices of exposure will sometimes be closed by a deposit of dentine; thinks the oxy-chloride of zinc or os-artificial will always kill the pulp; thinks capped pulps will always die.

Dr. Hill—Believes that dental pulps can and ought to be saved; it is wrong to destroy a nerve if it can be preserved.

If he produces an exposure by excavation, he depletes the pulp, simply puncturing it for that purpose. When it is healthy, fills at once; usually places a thin layer of Hill's stopping upon it, and then fills with gold. Succeeds in saving a large proportion of the pulps thus treated.

Dr. Clark—Asks if the pulp of a permanent central incisor of a child eight or nine years old should be at once destroyed, without an effort to save it.

In such cases, he cleanses out carefully, and makes some application to allay inflammation, if such a condition exists, and then fills lightly and carefully with tin; puts it in simply sufficient to exclude foreign substances. Sometimes repeats this operation a number of times, before the condition he wishes is brought about. By such treatment, will in a great many cases save the vitality of the teeth ; has filled teeth with gold, that Dr. Hayden had filled with tin, and properly too; considers these temperary fillings of tin an important matter.

Dr. Buckingham—Uses oxy-chloride of zinc for destroying the pulps. In filling, always prefers to cut to sensitive dentine.

Dr. Searle—Has had some hope that there is somewhere in nature some agent that will enable us to save pulps of teeth.

Dr. Colburn—Advises every one to prepare his own oxy-chloride of zinc, in order to have it pure.

Dr. J. A. Perkins—Trembles when he fills a tooth with an exposed pulp, and yet is as desirous to save them as any; thinks it important to do so.

Dr. Hill—Does not apply anything to the pulp after depleting, if it is healthy; thinks a wounded pulp will throw out a plasm for its own protection.

Dr. Pease—Have capped nerves in many cases since the meeting at Saratoga, but in every case they have died, and that in a short time. In many cases, where there is a thin layer of dentine over the pulp, the latter will die if the tooth is filled with gold.

Dr. Clark—Nature makes provision for the protection and preservation of the pulps, as is shown in teeth worn down beyond a point once occupied by the pulp, the chamber being perfectly filled with a secondary deposit.

Dr. J. A. Perkins—Considers tin better than gold for filling soft teeth ; thinks it especially applicable for temporary fillings for children's teeth.

Dr. PEASE—We can not produce the same condition by filling that nature does in the wearing down of the teeth by attrition. Thinks lead as good or perhaps better than tin for temporary fillings.

Dr. ATKINSON—Does not wish to be misunderstood; he *knows* of cases where pulps have been exposed, which have been protected, and secondary dentine closed up the orifice of exposure, and by this means the life of the tooth effectually saved. For caps, uses lamina of horn, saturated with creosote. Gave an interesting case, in which covering for a pulp was formed in a very short time.

In some remarks upon the importance of the dentist making frequent examinations, he recommended the Coddington lens, as the one best adapted for the dentist's use.

Dr. ALLEN—Will ossific deposit be made by the tooth pulp, after the development of the tooth? *Yes.* This fact may be verified by the examination of the teeth of many animals, as well as man. When a pulp is exposed, and healthy, this deposit will be made, if protected from irritating agencies; but in ordinary cases, the latter is so difficult to accomplish, that his practice usually is to remove the pulps and fill the fangs; has more confidence in this than in capping, with a view to save the life of the tooth.

Adjourned to meet to-morrow morning, at 8½ o'clock.

FOURTH DAY—MORNING SESSION.

Convention met according to adjournment, Vice-President in the chair.

The Minutes of yesterday afternoon's session were read, amended and adopted.

The subject of dental pulps was continued.

Dr. ROGERS—Suggests that the apparent success in saving exposed nerves or pulps might present a different phase if the cases were examined more frequently and more closely; let us always examine and see what the condition is with these cases. After exposure and inflammation, the tooth pulp

*can not* be saved. Children's teeth oftentimes require treatment different from that appropriate in the treatment of adult teeth.

Dr. STONE—Have capped and tried to save nerves for six years; have tried all methods, but usually failed; have occasionally saved a nerve, and secured a bony covering.

For the removal of pulps, prepare to excise them at once, rather than make any therapeutic application.

Dr. ROBERTS—Thinks there are many cases in which, by careful treatment, pulps of teeth may be saved; three-fourths of the cases treated for this purpose are failures; always preferred to excise the nerve, without medication. Uses platinum instruments for this purpose.

Dr. FRANKLIN—Uses a barbed instrument for removing pulps.

Discussions on this subject here closed.

Mechanical Dentistry was taken up for one hour by consent.

Dr. Franklin read an essay upon temporary dentures, (for which see next number of the Register.)

*Resolved*, That at 10 o'clock, twenty minutes shall be occupied in the exhibition of instruments, models, etc., at the expiration of which time the discussions on mechanical dentistry may be resumed.

Dr. ROBERTS—Inserts temporary teeth immediately after the removal of the natural teeth; by this course, there is less soreness of the gums, and the natural form of the parts is better preserved, and the use of teeth is not lost.

In making temporary dentures, let the edge of the plate come to the center of the ridge; use a small air chamber; metal plates are better than rubber, for even temporary work.

Dr. C. PALMER—Gold still lives to bless its friends; platinum is heavy, rubber is light; these will not meet all cases. He uses gold plate for restoring the contour of the face, by curving over the borders of the plate, to suit the case. He exhibited models and specimens of this style of work, which were very beautiful and interesting, but a full description would occupy too much space here. (We hope to have a description of the work from the Doctor's pen for the Register, ere long.—Rep.)

Dr. W. H. Allen—Refers to the fact that rubber plates sometimes produce a sense of heat and irritation of the mucous membrane upon which it rests; he asked for an explanation.

Dr. Taft—Remarked that metal plates will sometimes, though but rarely, irritate the mucous membrane on which they rest; but the difficulty referred to by Dr. Allen occurs far more frequently with rubber than with metal plates, and chiefly from the fact that it is so poor a conductor of heat—the heat does not escape from the mucous membrane covered by a rubber plate as when covered by a metal plate, or when uncovered, that alone will be sufficient in some cases to materially affect the parts. Referred to the difference in the conducting property of metals and rubber.

Dr. Stone—Asked if rubber plates will hold more heat than metal plates.

Dr. Taft—Made some farther remarks upon the conducting properties of different substances, with a view to answer Dr. Stone's query.

Dr. Whitney—Inserts temporary dentures immediately after the extraction of the natural teeth; lets the neck portion of the tooth pass slightly into the sockets of the natural teeth—this, of course, is the true location. Never uses air chambers in any case. Always uses rubber for temporary dentures. The patient may not use it more than six or nine months, but then make a new piece.

Dr. Clark—Thinks it wise to prove all things, and hold fast that which is good. He used to make double gold plates, some of which, he thinks, have not been excelled by anything. We might sometimes, with profit, return to old things.

The hour of 10 o'clock having arrived, twenty minutes were devoted to the exhibition of instruments and appliances.

Dr. House—Exhibited an instrument case, which was a "*multum in parvo*" indeed. It was quite a small case, and yet contained all the instruments for operating and the tools and appliances for the mechanical department. The room was all occupied.

Dr. Franklin—Exhibited a fusible gauge.

Dr. Whitney—Expressed some doubts as to the reliability of fusible gauges.

Dr. Snow—Exhibited a "wet nurse"* for keeping corundum wheels wet while grinding. It consists of a pint bottle, with a stopper with sufficient leakage; a small hole is made in the side of the bottle to admit air as the water escapes; a rubber band passes round the bottle and covers the hole; by raising this slightly, the admission of air and the escape of water can be regulated. The hole is readily made with a hard steel drill moistened with camphor and turpentine.

Dr. Rogers—Refers to Dr. Palmer's gold work, and particularly to the fact that the rim can be changed at any time and to almost any extent.

Dr. Burras—Can do anything in these respects with rubber that can be done with anything else. A gold plate should never be touched with pliers after it is stamped.

The introduction of subjects under the head of miscellaneous business was now in order.

Dr. Palmer—Uses single gum teeth, and puts on a band, thus forming sockets; to prevent springing, use fine gold plate, which prepare as follows: Take three sovereigns, and add ten cents of silver. Fit all parts of the work as perfectly together as possible, especially the linings to the plate; uses plaster and sand for investment; and in the investment puts a wire gauze frame. Begins the soldering at the posterior portion of the arch.

Dr. Roberts—Thinks Dr. Palmer's plumpers will retain food, and be difficult to keep clean.

Dr. Allen—Thinks temporary sets of teeth should be made as carefully and as perfectly as possible. Has made it

*Our name.—Rep.

an object to reach the highest point of excellence. Will not condemn any style of work. Usually puts in temporary sets within a week after extraction. Makes temporary sets of continuous gum work; takes the sockets of the natural teeth as a guide in the arrangement of the artificial substitutes. After this has been worn from nine months to a year, break off the body, and re-adjust the plate to a new cast. This can be done on the plaster cast with a mallet; do not always remove the body from palatine part of the denture; make these sets so perfect that they can scarcely be detected; always recommend a duplicate set. To prevent contraction, use a platina bar between the heels of the plate. Prefers solid plumpers. For straggling partial pieces, use gold plate. Made some remarks upon the principles involved in the restoration of the contour of the face. Referred to the subject of mending; suggested that it is as easy as anything else, when understood. In baking, heat twice for the body and once for the gum. Temper the work by letting it stay in the upper muffle over night.

Dr. Burras—Said that notwithstanding all this, he would use rubber all the time, and in all cases. Dr. B. also read an interesting paper on extraction of the teeth.

Dr. B. Wood—Presented a paper on Plastic Metallic Fillings, which, for want of time, was not read, but ordered to be published. (For which see page 400, present number of the Register.)

Dr. Hayes—The introduction of rubber has added another resource to the dental art, but we will use gold more after awhile.

Dr. Westcott—Expressed some fears of the influence arising from the indiscriminate use of rubber for artificial dentures.

Dr. House—Makes rubber work, and will make it as long as it is called for.

Dr. Taft—Expressed much regret at the state of things arising from the indiscriminate use of rubber work. It has become so cheapened that it brings this department of dental practice down to the level of the common tinker; it not only exercises a pernicious influence upon this department, but as great and injurious an influence upon the operative department also. Many reason on this wise, If artificial teeth are made so cheap and as good as represented, I will not be at the expense and pains of having my natural teeth filled. I will let them go, since I can supply their place so easily. Reasoning of this kind influences the great mass of the people, and it is difficult to meet it, not because it is not fallacious, but because it is specious.

The present state of affairs in regard to this matter tends to discourage all our best operators, because their cherished and best efforts for the elevation of the profession are thereby frustrated. How shall this growing evil be met? Let every one take this problem for investigation, and action too.

Dr. Westcott—Took up this subject, and continued at some length upon it, urging it upon the profession to correct this matter, as they only have the power to do it.

On motion,

*Resolved*, That the thanks of the Convention be tendered to the reporters.

On motion,

*Resolved*, That the sum of $20 be paid Dr. F. H. Norton for preparing the reports for the newspapers.

The Executive Committee presented the following programme of subjects for discussion at the next annual meeting viz.:

1st. Causes influencing an abnormal development of the teeth.

2d. Treatment of Dental Irregularity, and appliances for the same.

3d. Filling teeth—Filling temporary teeth—Best material for the same.

4th. Diseases of the antrum, and treatment.

5th. Treatment of cleft palate.

6th. Alveolar Abscess.
7th. Mechanical Dentistry.
8th. Miscellaneous Business.

W. H. DWINELLE,
WM. A. PEASE,
D. W. STONE,
D. W. PERKINS,
T. L. BUCKINGHAM,
*Ex. Committee.*

Adjourned to meet at Saratoga on the first Tuesday of August, 1863.

---

